# Quantitative proteomics analysis of differentially expressed proteins induced by astragaloside IV in cervical cancer cell invasion

**DOI:** 10.1186/s11658-020-00218-9

**Published:** 2020-03-31

**Authors:** Chenglai Xia, Zhihong He, Yantao Cai

**Affiliations:** 1 Foshan Maternal and Child Health Research Institute, South Medical University Affiliated Maternal & Child Health Hospital of Foshan, 11 Renmin Xi Street, Foshan, 528000 China; 2 Department of Dermatology and Pheumatology, South Medical University Affiliated Maternal & Child Health Hospital of Foshan, 11 Renmin Xi Street, Foshan, 528000 China

**Keywords:** Astragaloside IV, Quantitative proteomics, iTRAQ, Parallel reaction monitoring, Cervical cancer

## Abstract

**Background:**

Cervical cancer remains the second leading cause of mortality in women in developing countries. While surgery, chemotherapy, radiotherapy, and vaccine therapy are being applied for its treatment, individually or in combination, the survival rate in advanced cervical cancer patients is still very low. Traditional Chinese medicine has been found to be effective in the treatment of cervical cancer. Astragaloside IV (AS-IV), a compound belonging to *Astragalus* polysaccharides, shows anticancer activity through several cell signaling pathways. However, the detailed molecular mechanism governing the anticancer activity of AS-IV remains unknown.

**Material and methods:**

In our study, we performed tumor xenograft analysis, transwell cell migration and invasion assay, Western blot analysis, and iTRAQ combination by parallel reaction monitoring (PRM) analysis to study the molecular mechanism of AS-IV in the suppression of cervical cancer cell invasion.

**Results:**

Our results showed that AS-IV suppressed cervical cancer cell invasion and induced autophagy in them, with the tumor growth curve increasing slowly. We also identified 32 proteins that were differentially expressed in the SiHa cells when treated with AS-IV, with 16 of them involved in the upregulation and 16 in the downregulation of these cells. These differentially expressed proteins, which were predominantly actin–myosin complexes, controlled cell proliferation and cell development by steroid binding and altering the composition of the cell cytoskeleton. DCP1A and TMSB4X, the two proteins regulating autophagy, increased in cervical cancer cells when treated with AS-IV.

**Conclusions:**

We conclude that AS-IV could inhibit cervical cancer invasion by inducing autophagy in cervical cancer cells. Since iTRAQ combination by PRM has been observed to be useful in identifying macromolecular target compounds, it may be considered as a novel strategy in the screening of anticancer compounds used in the treatment of cervical cancer.

## Introduction

The cancer data for 2019 reveal that cervical cancer continues to be the second leading cause of mortality in women, particularly in those between 20 and 39 years of age [1]. About nine cancer patients are observed to die from cervical cancer every week [2]. While surgery, chemotherapy, radiotherapy, and vaccine therapy are applied for its treatment, individually or in combination, the survival rate in advanced cervical cancer patients is still very low, and the side effects of the therapies are continuing to increase [3, 4]. In addition, the 2-, 4-, and 9-valent HPV vaccines do not show any clinical effectiveness in the treatment of cervical cancer [5–7]. Therefore, there is an urgent need to explore more effective drugs to prevent cervical cancer cell invasion and improve the quality of life in cervical cancer patients.

Several anticancer drugs have been derived from natural products [8]. There are about 350,000 plant species on earth, which have been a major source of medicines for a long time. The Chinese began to use herbs to treat cancer, as well as cardiovascular and other incurable diseases, several thousands of years ago [9, 10]. *Astragalus* roots which have been used in traditional Chinese medicine include those of *Astragalus mongholicus* and *A. membranaceus* [11]. *Astragalus* has the effect of invigorating *qi* for strengthening superficies, diuresis, platoon poison, discharging pus and accumulating sore and muscle [12]. Astragaloside IV (AS-IV), a lanolin alcohol-form tetracyclic triterpenoid saponin, is an active ingredient of the Chinese herb *Astragalus* [13]. Researchers have found that AS-IV showed anticancer effects by targeting the JNK/c-Jun/AP1, Akt/GSK-3β/β-catenin, PI3K/Akt/NF-κB, and other signaling pathways [12, 14–17]. However, the detailed molecular mechanism governing the anticancer activity of AS-IV remains unknown. Therefore, in this study, iTRAQ proteomics was used to identify the differentially expressed proteins in cervical cancer cells treated by AS-IV and aim to discover in detail the molecular mechanism governing the anticancer activity of AS-IV.

## Methods and materials

### Cells, antibodies and siRNA

SiHa and HeLa cells, two kinds of human cervical cancer cell lines, were obtained from Dr. Xia’s laboratory (Foshan, China). Fetal bovine serum was bought from Thermo Fisher Scientific (Carlsbad, CA, USA). AS-IV (molecular weight, 784.97) was obtained from Sigma-Aldrich Corporation (St Louis, MO, USA). The antibodies rabbit anti-human LC3I/II, GAPDH, Atg7, and Atg12, and their corresponding secondary antibodies were acquired from Cell Signaling Technology Inc. (Beverly, MA, USA). DCP1A siRNA and TXSB4X siRNA were purchased from Sangon Biotech Co., Ltd. (Shanghai, China).

DCP1A siRNA sequence: sense (5′-3′), GACACAACCACUUGGGAAATT; antisense (5′-3′), UUUCCCAAGUGGUUGUGUCTT.

TXSB4X siRNA sequence: sense (5′-3′), AGACAGAGACGCAAGAGAATT; antisense (5′-3′), UUCUCUUGCGUCUCUGUCUTT.

### Cell culture

The SiHa and HeLa cells were cultured in Dulbecco’s modified eagle medium (DMEM) containing 10% fetal bovine serum (Invitrogen, Carlsbad, CA, USA) and 1% streptomycin (Sigma-Aldrich, St. Louis, MO, USA). Cells (1 × 10^7^) were harvested by trypsinization after treatment with 25 μM astragaloside IV or not for 12 h and washed 5 times by using of PBS.

### Western blot analysis

Based on our previous study [18], cell total protein samples were taken from a − 80 °C refrigerator. After the addition of lysis buffer, the samples were centrifuged at 12,000 *g* at 4 °C for 10 min. The supernatants were transferred to new centrifuge tubes and quantified using a BCA kit (Novagen, MA, USA). The precooled protein samples and predyed markers were added to the gel. After running the gel, protein was transferred onto the PVDF membrane (Millipore, MA, USA). The non-specific antigens on the PVDF membrane surface were blocked by 5% non-fat milk. After hybridization with the corresponding primary and secondary antibodies and washing with Tris-buffered saline (0.1% Tween20), the PVDF membrane was developed using an enhanced chemiluminescence solution (Pierce, IL, USA) and subsequently photographed using a Bio-Rad gel imaging system.

### Cancer cell invasion analysis

Based on our previous study [18], 24-well transwell invasion chambers (Corning, NY, USA) were used to analyze the cancer cell invasion. The matrix gel (1 mg/ml; Bio-Rad, CA, USA) was introduced into the upper chamber and the cell culture medium containing 10% fetal bovine serum was introduced into the lower chamber; 2 × 10^5^ per-well HeLa or SiHa cells were seeded into the upper chamber. After treatment with AS-IV for 12 h, the cells that had invaded the lower chamber were fixed with methanol and dyed using crystal violet. We used an inverted microscope to count the number of invaded cells.

### iTRAQ proteomics

Based on a previous study [19], the labeling agent of the iTRAQ (Thermo, USA) kit was dissolved in acetonitrile buffer after thawing. Briefly, cell samples were harvested in lysis buffer (8 M urea and 1% protease inhibitor cocktail) and sonicated three times on ice with a high-intensity ultrasonic processor (Scientz, China). The protein supernatant concentration was measured with a BCA kit after centrifugation at 12,000 *g* at 4 °C for 10 min according to the manufacturer’s instructions. Three hundred micrograms of protein in pre-samples was subjected to analysis. The peptide was lysed with trypsin and desalted with Strata X C18, drying in a vacuum container. The dried peptide was labeled by mixing with a labeling agent for 2 h at room temperature, desalted, and lyophilized in a vacuum container. Then, the tryptic peptides were fractionated using a C18 column with 5 μm particles, 4.6 mm ID and 250 mm length (Thermo Betasil) equipped in a high pH reverse-phase HPLC machine.

The tryptic peptides were dissolved with 0.1% formic acid and were subjected to an nanospray ionization (NSI) source followed by tandem mass spectrometry (MS/MS) in Orbitrap Fusion Lumos (Thermo, USA) coupled online to the UPLC. 2.0 kV of electrospray voltage was applied and 350 to 1550 of m/z scan range was applied for a full system scan. After being detected at a resolution of 60,000 or 15,000 in the Orbitrap, intact peptides or fragments were selected for MS/MS using the NCE setting of 28. The data-dependent procedure alternated between one MS scan followed by 20 MS/MS scans with 30.0 s dynamic exclusion. Automatic gain control (AGC) was set at 5E4. Fixed first mass was set as 100 m/z. The resulting MS/MS data were processed using the Maxquant search engine (v.1.5.2.8). In a brief, tandem mass spectra were searched against the human UniProt database (Human_SwissProt_1808) concatenated with the reverse decoy database. Allowing up to 2 missing cleavages, trypsin/P was considered as the cleavage enzyme. The mass tolerance for precursor ions was set as 20 ppm in first search and was set as 5 ppm in the main search. 0.02 Da was set as the mass tolerance for fragment ions. FDR was adjusted to less than 1% and the minimum score for modified peptides was set to more than 40. The peptide was identified by its second-order spectrum and quantified by its intensity. FDR was adjusted to less than 1% and the minimum score for modified peptides was set to more 40. The mass error (ppm) needed to be within ±10.

### Protein function enrichment and cluster analysis

Based on a previous study [20], collecting the protein enrichment information classified with different functions and corresponding *P*-values, we identified a significant enrichment functional classification (*P* < 0.05). At first the screened P-value data matrix was logarithmically transformed (−log10 model), then the transformed data matrix was used to classify the functions by Z transform, and finally the hierarchical clustering (Euclidean distance, average connection clustering) method was used for unilateral clustering analysis of the dataset obtained after Z transformation. A heatmap with the R language package gplots was drawn to show the cluster relation.

### Protein–protein interaction and network analysis

Based on a previous study [20], differentially expressed proteins screened from different comparison groups were numbered and the protein–protein interactions were identified using the STRING (v.10.5) protein network database. Based on the confidence scores (a score of 0.7 was considered as a high confidence score), the differential protein interactions were extracted and the corresponding protein–protein interaction networks were visualized using the network D3 package.

### PRM analysis

Based on the method mentioned by Sun BB [21], dithiothreitol and iodide acetamide were added to the protein solution. The mixture was hydrolyzed overnight at 37 °C and the ratio of trypsin:protein was 1:50. The hydrolysis was continued for 4 h and the ratio of trypsin:protein was 1:100. The peptides were dissolved in 0.1% (v/v) aqueous formic acid, and the mobile phase was isolated using the EASY-nLC1000 high-performance liquid chromatography system (Thermo Scientific, Carlsbad, CA). The isolated peptides were subjected to an NSI source followed by mass spectrometry using the Q Exactive Plus Hybrid Quadrupole-Orbitrap (Thermo Scientific) mass spectrometer. The tandem mass spectrometry (MS/MS) data were processed using Skyline (v.3.6). Full details are given in Supplementary Methods.

### Animal experiments

Four-week-old BALB/c nude mice weighing 15–17 g were provided by the Experimental Animal Center of Guangdong Province (SCXK No. SCXK [yue] 2006–0015) and fed in specific pathogen-free animal rooms. All animal experimental protocols were approved by the Medical Ethics Committee of Southern Medical University affiliated Maternal & Child Health Hospital of Foshan City (Guangdong Province, China). The temperature and the humidity in the feed room were 22–25 °C and 40–60%, respectively. The light and dark alternating times were 12 h/12 h. A SiHa cell suspension (0.1 mL; approximately 5 × 10^6^ cells) cultured using serum-free medium was injected into the nude mice left fore region near the axilla. Subcutaneous tumor volumes from the mice were measured using an electronic vernier caliper. Mice with tumor diameter 0.3–0.5 cm were numbered and randomly divided into 3 groups with 5 mice in each group. AS-IV (25 mg/kg/d) was gavaged into the mice. The growth data of the tumor were record twice a week and the tumor volume was calculated using the following formula: V = 0.5 × A × B^2^, where V is the tumor volume, A is the longest diameter of the tumor, and B is the shortest diameter of the tumor. The mice were sacrificed and photographed after 35 days. All experimental procedures were approved by the Institutional Animal Care and Use Committee of South Medical University Affiliated Maternal & Child Health Hospital of Foshan (Foshan, China).

### Statistical analysis

The SPSS16.0 software (IBM SPSS, Chicago, IL, USA) was used for data analysis in this study. Student’s t-test and one-way ANOVA followed by Dunnett’s test, at 5% probability level, were applied for data of morphological and mutagenic assessments. The significance of differences was assessed by Student’s t-test and one-way analysis of variance (ANOVA) followed by Dunnett’s test, and *P* values less than 0.05 were considered statistically significant.

## Results

### Inhibition of cervical cancer cell proliferation, invasion and xenograft tumor growth by AS-IV

**Fig. 1:**
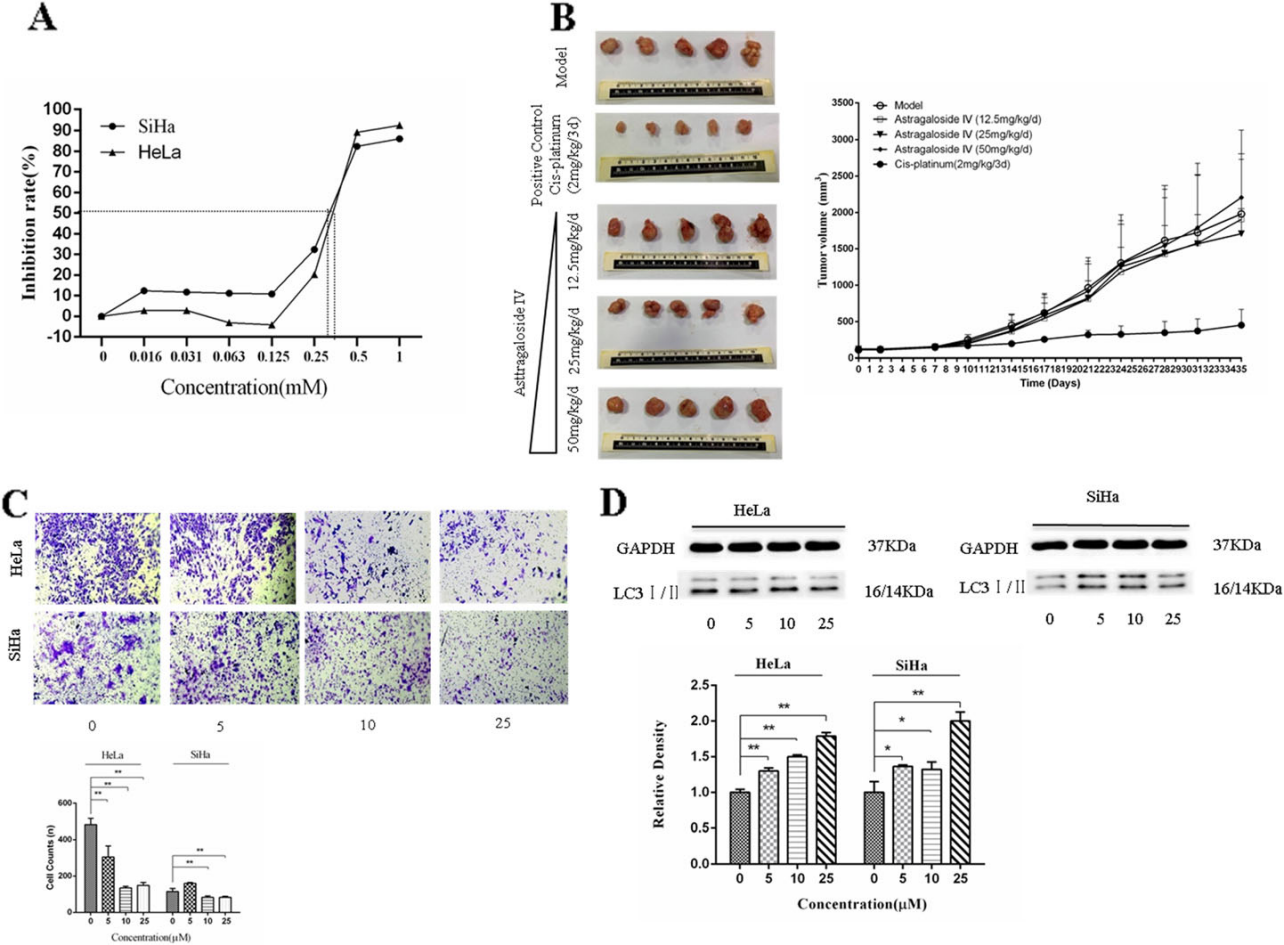
AS-IV inhibits cervical cancer cells invasion and xenograft tumor growth in xenograft models. **a** CCK-8 assay detected proliferation of SiHa and HeLa cells treated with different concentrations of AS-IV for 72 h. **b** The xenograft model of cervical cancer was generated by injecting SiHa cell lines into BALB/c nude mice. AS-IV (a dose of 12.5 mg/kg/d, 25 mg/kg/d, 50 mg.kg/d) was applied for tumor rescue in mice for 35 days by gastric gavage and the inhibition of tumor growth by AS-IV was evaluated. **c** HeLa and SiHa cell invasion was examined by transwell analysis in the present of AS-IV of different concentrations treated for 12 h. **d** Expression of LC3I/II in HeLa and SiHa cells was measured by western blot in the presence of AS-IV of different concentrations treated for 12 h. Data are presented as mean ± standard deviation, *n* = 3. *P < 0.05 and **P < 0.01 refers to the 0 μM AS-IV control group

Autophagy, a process of phagocytosis of the cytoplasmic proteins or organelles and their inclusion into vesicles, fusion with lysosomes to form autophagy lysosomes and degradation of the contents they contain, can inhibit inflammation caused by cancer cells and stabilize cell chromosome construction [22, 23]. AS-IV, an *Astragalus* polysaccharide with a distinct structure, is present in *Astragalus* in smaller amounts when compared with other polysaccharides [24]. We treated cervical cancer cells with AS-IV for 72 h followed by the analysis of cell proliferation with CCK-8. As shown in Fig. 1a, AS-IV directly inhibited the proliferation of HeLa and SiHa cell lines infected by HPV-18 or HPV-16 respectively. The IC_50_ values of AS-IV for inhibiting cervical cancer cells were 0.49 ± 0.03 mM and 0.27 ± 0.03 mM in HeLa and SiHa cells, respectively. However, both the antivirus and anticancer effects of AS-IV are higher than those of the other *Astragalus* polysaccharides [25, 26]. In our study, we used AS-IV (12.5 mg/kg/d, 25 mg/kg/d, 50 mg/kg/d) to treat human cervical cancer xenografts in mice, and chose cisplatin as a positive drug to treat cancer. After 35 days of treatment, the tumor volume in the cisplatin treatment group had obviously shrunk when compared to that in the PBS-treatment group (Fig. 1b). It is, therefore, inferred that our anticancer drug treatment system was effective in the treatment of cervical cancer. Moreover, the tumor volume in the AS-IV (25 mg/kg/d) treatment group decreased compared to the PBS-treatment group. We used AS-IV (0, 5, 10, 25 μM) to treat HeLa and SiHa cells. After 12 h of treatment with AS-IV, we found that the cancer cell invasion was inhibited in the HeLa and SiHa cells (Fig. 1c). LC3I/II, a protein marker of autophagy, was induced in the HeLa and SiHa cells (Fig. 1d). These results showed that AS-IV inhibited the growth of cervical cancer cells, especially inhibiting the growth of HPV-16-infected human cervical cells, and inhibited xenograft tumor growth by inducing autophagy.

### Analysis of differentially expressed proteins by iTRAQ

**Fig. 2:**
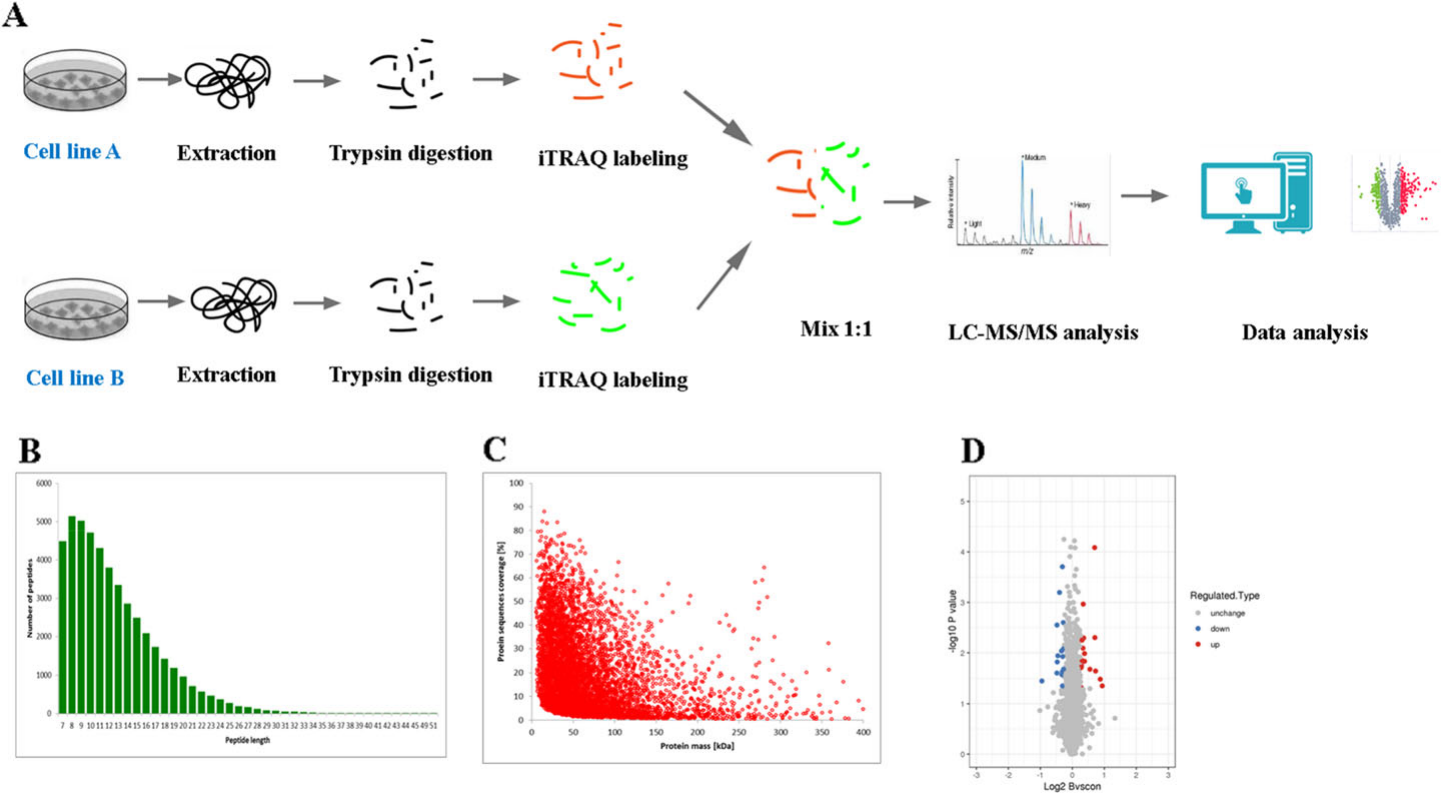
Differentially expressed proteins were evaluated by iTRAQ proteomics. **a** Flow diagram of iTRAQ proteomics. **b** Length distribution of the peptides identified by mass spectrometry. **c** Relationship between protein molecular weight and coverage. **d** Volcano plot of differentially expressed proteins

In order to understand in detail the mechanism of cell autophagy induced by AS-IV to suppress inflammation in the progression of cervical cancer, we analyzed the differential protein expression in AS-IV-treated cervical cancer cells using the iTRAQ combination. Peptides digested by trypsin were analyzed using high-resolution mass spectrometry (Fusion Lumos). The primary ions and secondary fragments of the peptides were also analyzed using high-resolution mass spectrometry (Fusion Lumos). All obtained data were analyzed through a certain bioinformatics database (Fig. 2a). Most of the peptide fragments were found to contain 7–20 amino acid residues, an observation that conforms with the general rules of trypsin enzymatic hydrolysis and HCD fragmentation (Fig. 2b). There was a negative correlation between molecular weight and the protein sequence coverage. Theoretically, larger molecular weight proteins can produce more enzymatic fragments to achieve the same coverage. That is to say, it needs more peptides identified from a large protein to achieve the same coverage. The length of the peptide fragments identified meets the quality control requirements (Fig. 2c). In this experiment, we identified 6964 proteins, of which 6072 proteins were quantified. Considering 1.2-fold expression as the standard we found that 32 proteins were differentially expressed when the SiHa cells were treated with 25 μM AS-IV for 12 h. Out of these 32 differentially expressed proteins, the expression of 16 proteins was upregulated and that of the other 16 was downregulated (Fig. 2d).

### Functional classification of differentially expressed proteins in AS-IV-suppressed cervical cancer cells

**Fig. 3:**
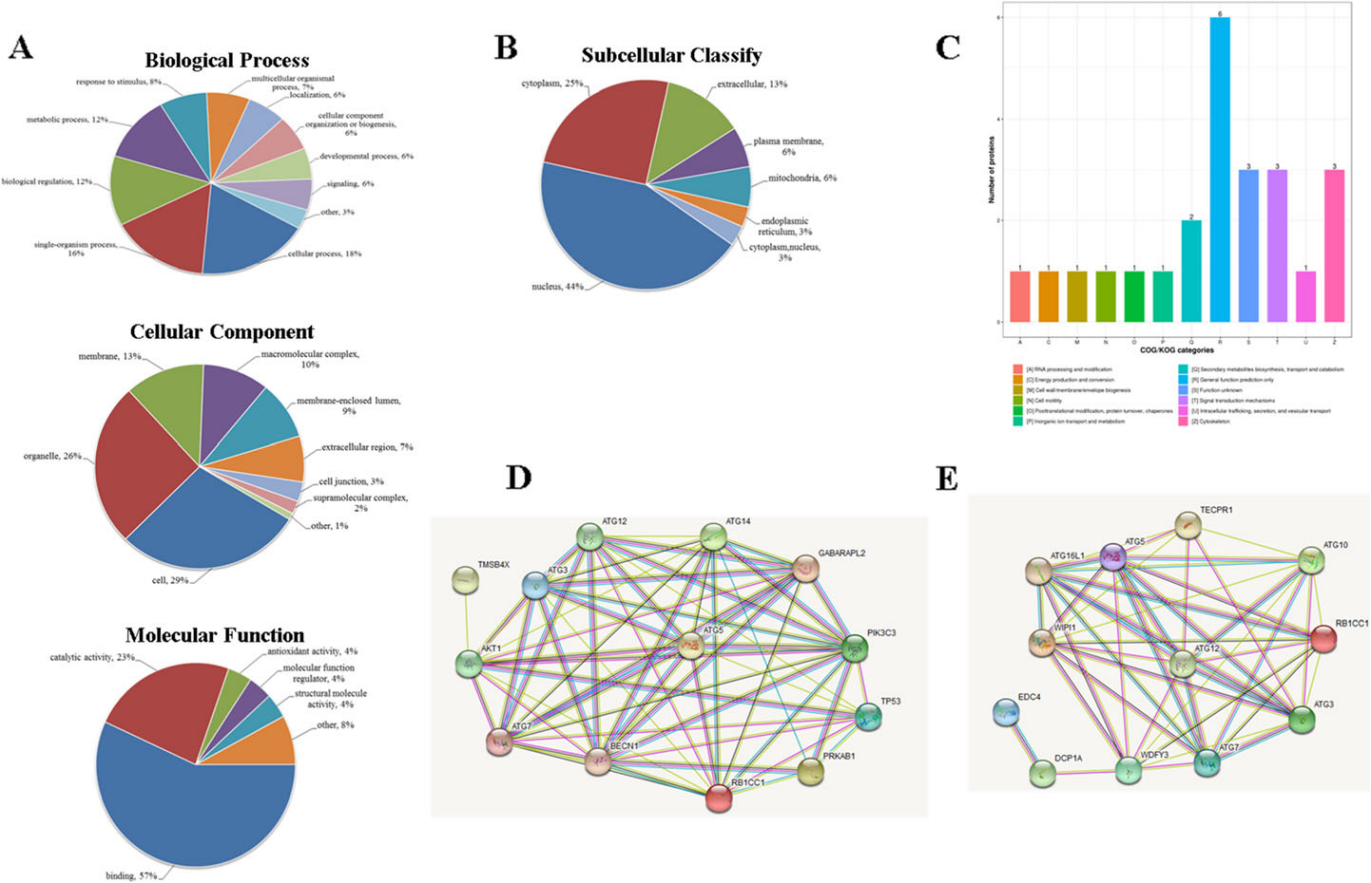
Differentially expressed proteins under the presence of AS-IV treatment in cervical cancer cells analyzed by Gene Ontology. **a** BPs, CCs, and MFs evaluated. **b** Subcellular localization and classification of differentially expressed proteins. **c** Clusters of orthologous group classification of differentially expressed proteins. **d** TMSB4X/Akt/Atg5/Atg12 pathway; **e** DCP1A/WDFY3/Atg12 pathway. Proteins are represented by colored spheres, and interaction proteins are represented by white spheres. The interactions already recorded in the database are shown with blue lines and the interactions that are confirmed by our results are shown in purple. Two adjacent genes are linked by green lines and gene fusion is shown with red lines

Cluster analysis, based on the functional enrichment of the differentially expressed proteins, could analyze the potent relationship or distinction between the proteins based on the Gene Ontology (GO) Database, Kyoto Encyclopedia of Genes and Genomes (KEGG) pathway, and their protein domains [25, 27]. GO is an important bioinformatics tool for analyzing the attributes of genes and genetic products, including their biological processes (BPs), cellular components (CCs), and molecular functions (MFs). In our study, we analyzed the distribution of differentially expressed proteins in the secondary annotation of GO. As shown in Fig. 3a, most differentially expressed proteins following the treatment with AS-IV to suppress cervical cancer invasion take part in BPs such as cellular (18%) and single-organism (26%) processes, biological regulation (12%), metabolic process (12%), and response to stimuli (8%). Similarly, differentially expressed proteins following the treatment with AS-IV to suppress cervical cancer invasion belong to cell (29%), organelle (26%), membrane (13%), and macromolecular (10%) complexes as part of CCs (Fig. 3a). As shown in Fig. 3a in MFs, differentially expressed proteins following the treatment with AS-IV to suppress cervical cancer invasion exhibit MFs such as binding activity (57%), catalytic activity (23%), antioxidant activity (4%), and MF regulator activity (4%).

InterProScan (http://www.ebi.ac.uk/interpro/) was utilized to analyze the subcellular classification of the differentially expressed proteins. As shown in Fig. 3b, out of the 32 proteins, 8 are located in the cytoplasm, 4 in the extracellular matrix, 14 in the nucleus, 2 in the plasma membrane, 2 in the mitochondria, and one each in the cytoplasm and endoplasmic reticulum. We also analyzed the clusters of orthologous groups of proteins (COG) of these differentially expressed proteins. As shown in Fig. 3c, out of the 32 proteins, there are 3 signal transduction proteins, 1 RNA processing and modification protein, 1 cell wall biogenesis protein, 1 cell motility protein, 3 cytoskeleton proteins, 1 intracellular trafficking, secretion, and vesicular transport protein, 1 molecular chaperone, 1 energy production protein, 1 inorganic ion transport and metabolism protein, 2 secondary metabolite biosynthesis proteins, 6 general function prediction proteins, and 3 unknown-function proteins. Protein–protein interaction network, a useful tool for analyzing key genes, has been applied in many bioinformatics studies [28]. STRING, a database of known and predicted protein–protein interactions, has been widely used in predicting the interactions between known and unknown proteins. As shown in Fig. 3d and e, we found that AS-IV upregulated Atg12 and induced cancer cell autophagy through DCP1A and TMSB4X. The results suggested that the progression of AS-IV in the suppression of cervical cancer invasion is related to cell signaling transduction, secondary metabolite biosynthesis, and transmembrane transport, and especially might target DCP1A and TMSB4X.

### Functional enrichment of differentially expressed proteins in AS-IV-suppressed cervical cancer cells

**Fig. 4:**
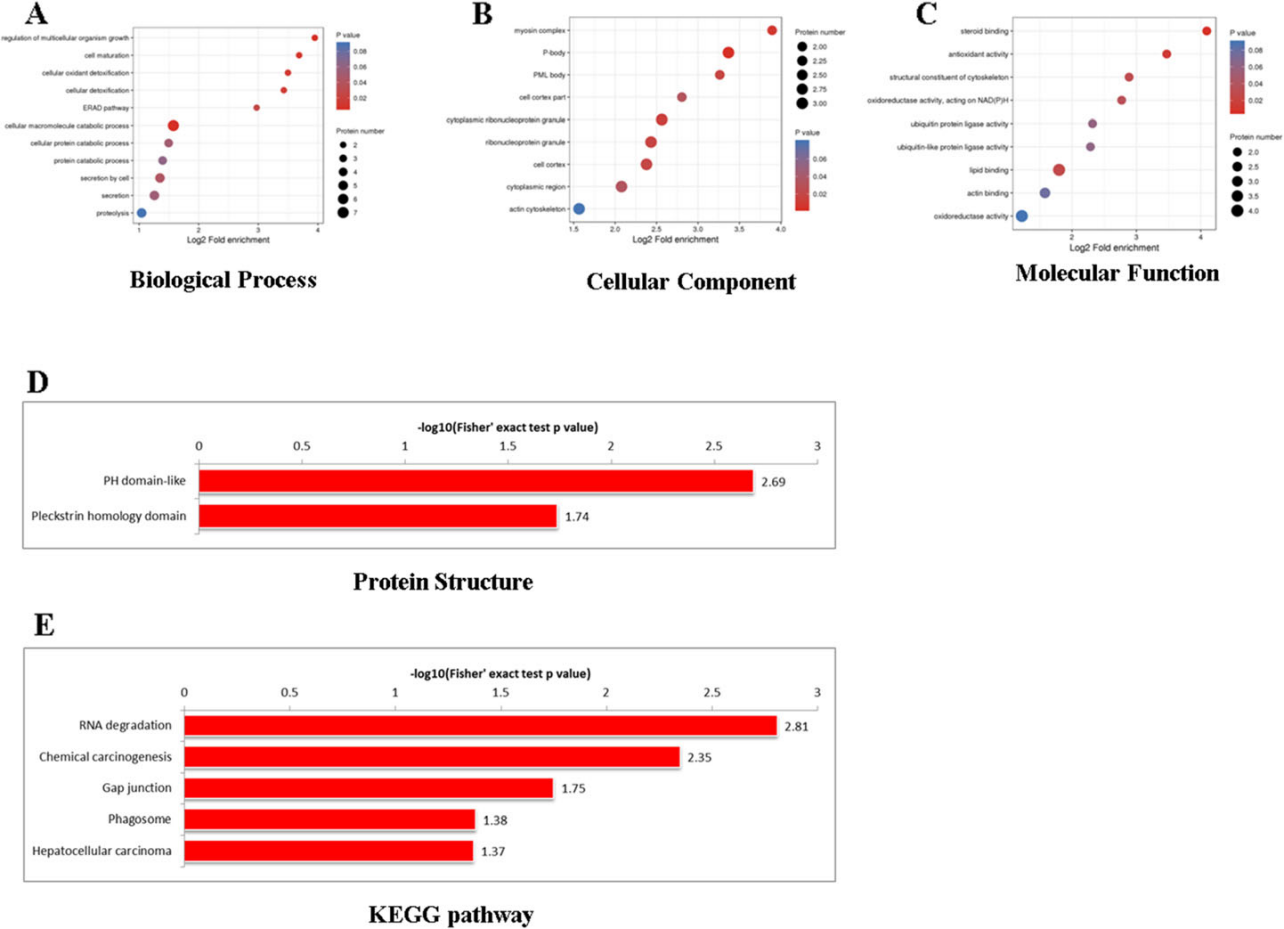
KEGG, GO and protein domain enrichment analysis of differentially expressed proteins. **a** biological processes; **b** cellular components; **c** molecular functions; **d** protein domain; and **e** KEGG pathway enrichment. The functional classification or pathway is shown in the vertical axis of the bubble chart; the proportion of the differentially expressed protein in the functional type divided by the ratio of the identified proteins was converted using log2 and shown on the horizontal axis. The circle color indicates the enrichment *P*-value and the circle size indicates the number of differentially expressed proteins in the functional class or pathway

Based on the protein notes, we analyzed the functional enrichment of the differentially expressed proteins at three levels: GO classification, KEGG pathway, and protein domain. We obtained the *P*-value using Fisher’s exact test and showed the functional classification or pathway of differentially expressed protein enrichments through a bubble diagram. The vertical axis of the bubble diagram represents the functional classification or pathway and the horizontal axis represents the ratio of differentially expressed proteins to the whole identified proteins, converting from log2. The color of the cycle represents the significant enrichment P-value, and its periphery represents the number of differentially expressed proteins in the pathway. Most of the differentially expressed proteins are involved in BPs such as cell growth and cell development regulation (Fig. 4a), and the CC of a majority of them are actin–myosin complexes (Fig. 4b). Similarly, the differentially expressed proteins perform MFs such as steroid binding, antioxidant activity, and altering the composition of the cell cytoskeleton (Fig. 4c).

The KEGG pathway is used to analyze the interactions among known MFs such as metabolic pathways, formation of complexes, and biochemical reactions. A protein domain, which consists of 25 to 500 amino acid residues, is a repeat sequence occurring during the progression of protein evolution. As may be seen in Fig. 4d, the differentially expressed proteins are enriched in chemical carcinogenesis and the RNA decay signal pathway. As shown in Fig. 4e, the proteins are largely PH domains interacting in nature. The results show that AS-IV regulates several cell-growth proteins, thereby suppressing cervical cancer cell growth.

### Induction of autophagy and suppression of cervical cancer invasion by AS-IV by targeting DCP1A and TMSB4X

**Fig. 5:**
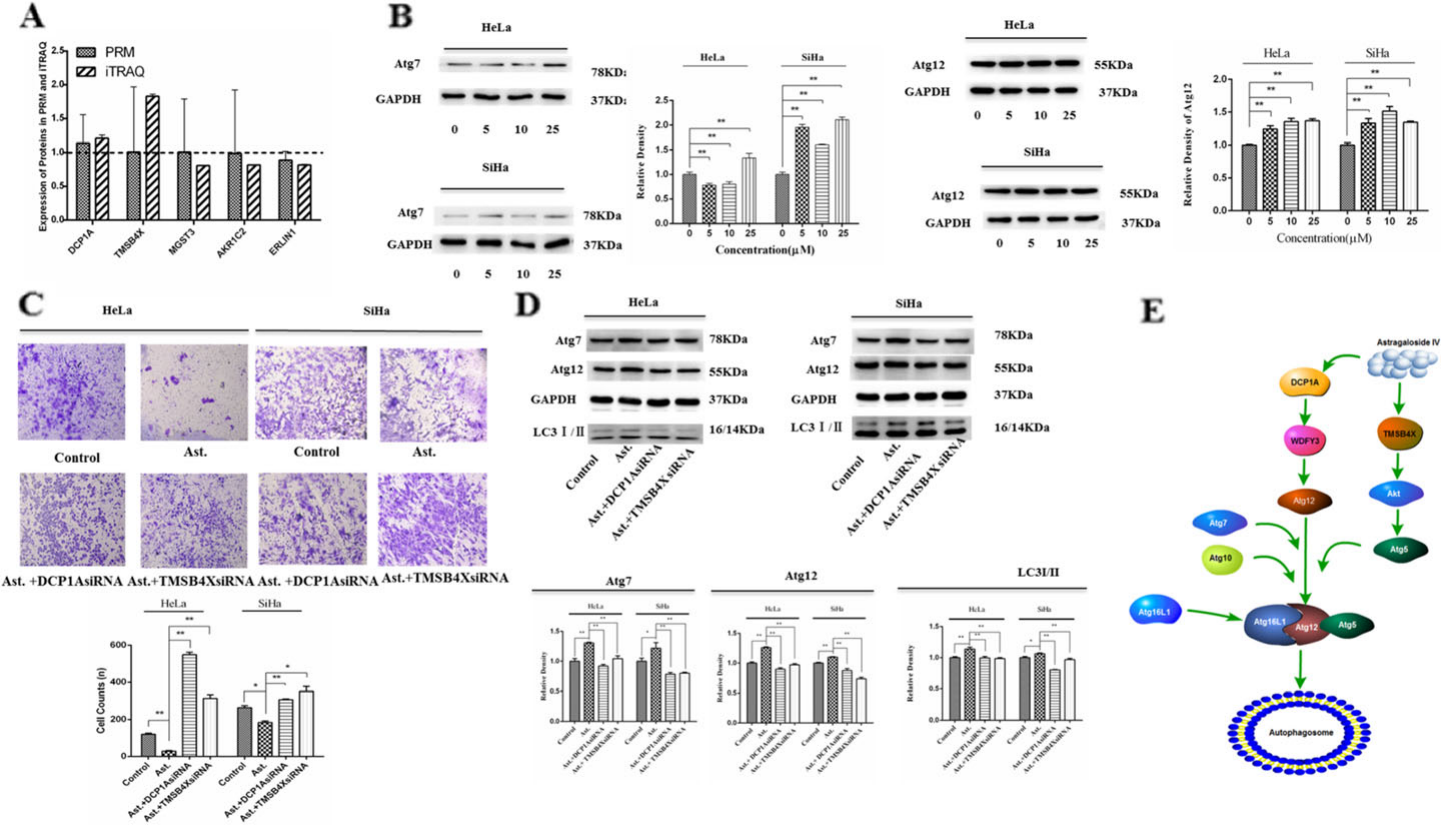
AS-IV induces autophagy and suppresses cervical cancer invasion by targeting DCP1A and TMSB4X. **a** Differentially expressed protein quantification by mass spectrometry-based targeted proteomics (PRM). **b** Expression of Atg7 and Atg12 in SiHa and HeLa cells was measured by western blot in the presence of AS-IV of different concentrations treated for 12 h. **c** SiHa and HeLa cells were treated with AS-IV in the presence of DCP1A siRNA or TMSB4X siRNA for 12 h and cell invasion was measured by transwell chamber culture. **d** SiHa and HeLa cells were cultured with AS-IV in the presence of DCP1A siRNA or TMSB4X siRNA for 12 h, and the expression of Atg7, Atg12, and LC3I/II was measured by western blot. **e** Mechanism map of AS-IV inducing autophagy through the TMSB4X/Akt/Atg5/Atg12 and DCP1A/WDFY3/Atg12 pathways. Results are presented as mean ± standard deviation, *n* = 3. *P < 0.05 and **P < 0.01 in comparison to the control group (0 μM AS-IV or AS-IV treated group)

PRM analysis, a principal method used to verify the proteomics data, selected specific peptides or target peptides and quantified the target protein or modified the peptides [29]. In the present study, we analyzed 5 of the 32 differentially expressed proteins using PRM instead of the traditional western blot. The results of the iTRAQ analysis showed that the expression of DCP1A and TMSB4X increased, whereas that of MGST3, AKR1C2, and ERL1N1 decreased (Fig. 5a). We observed the same results after the PRM analysis also. The quantitative information of PRM protein is calculated from the ion peak area of the peptide fragment (Supplement Table 1). We also measured the expression of Atg7 and Atg12, the key proteins involved in the autophagy process, in the presence of AS-IV. As shown in Fig. 5b, the expression of Atg7 and Atg12 increased in the presence of AS-IV. Meanwhile, as shown in Fig. 5c, we found that DCP1A siRNA or TMSB4X siRNA could rescue the AS-IV-induced inhibition of cell invasion. We also measured the expression of LC3I/II, Atg7, and Atg12 in the HeLa and SiHa cells. As shown in Fig. 5d, we found that AS-IV could upregulate the expression of LC3I/II, Atg7, and Atg12. However, the expression of LC3I/II, Atg7, and Atg12 decreased in the presence of AS-IV and DCP1A siRNA or TMSB4X siRNA. We also drew a mechanism map of AS-IV inducing autophagy through the Atg7/Atg12 pathway (Fig. 5e). Thus, DCP1A and TMSB4X may be regarded as the targets of cervical cancer treatment. These results suggest that AS-IV could target the proteins DCP1A and TMSB4X and induce autophagy, resulting in the suppression of cervical cancer proliferation and invasion.

## Discussion

In recent years, the identification of drug targets is the key challenge faced by practitioners in the field of precision pharmacology [30–32]. It is, therefore, necessary to establish a precision pharmacology system. However, the detailed pathological or pharmacological mechanisms that were inferred from related signaling pathways could not completely explain the molecular mechanism governing the anticancer activity of certain drugs [33, 34]. Since the physiological conditions are diverse and the signaling pathways are complex in vivo, several drug side reactions may occur if the drug targets are inaccurate, thereby causing drugs to work less effectively [35]. In the precision medicine and pharmacy era, it is, therefore, beneficial to optimize the physicochemical properties and action targets in terms of spatial and temporal distributions and action intensity as an effort to maximize the specificity of drug effects, reduce uncontrollable side effects, and improve the efficiency of research and development of specific therapeutic drugs for specific indications. During the occurrence and progression of diseases, drugs perform their functions mainly by targeting proteins and multiple signaling pathways they participate in. Proteomics, the study pertaining to protein products encoded and expressed by genes, is the way of understanding the interactions of protein molecules in the human body. Proteomics can, thus, reflect the dynamic changes in the physiological and pathological activities in the bodies [36]. The technology is currently being applied to understand several life phenomena that are unable to be explained at the genetic level. The proteomics of cells or bodies under normal conditions, diseases, and during drug treatment helps in the study of the molecular mechanism of the action of macromolecules and the identification of new drug targets, drug development, and clinical treatment guidelines [37]. iTRAQ combination by PRM is applied to identify the protein targets of macromolecules. First, the four-stage bar mass analyzer which has a selective detection capability is used to selectively detect the parent ion information of the target peptide segment. Then the samples are scattered in the HCD collision pool. Finally, all the fragments are transferred into the selected parent ion window and analyzed using the Orbitrap analyzer for high resolution and high accuracy. Thus, the target proteins or peptides in complex samples are accurately analyzed using the PRM system [38]. The technique, a combination of the high selectivity of a four-stage bar, high resolution, and high precision characteristics of the Orbitrap, shows its characteristic advantages including high-resolution sub-ion monitoring, wide second-level full scanning with its linear range up to 5–6 orders of magnitude, and simultaneous qualitative and quantitative analysis [39].

AS-IV may be categorized as a macromolecular compound because its molecular weight (784.97) is more than 500. In our study, we performed the iTRAQ combination using PRM to study the potential molecular mechanism of the suppression of cervical cancer cell invasion by AS-IV. We have considered the differential expression multiple of > 1.2-fold as the criterion for differential protein screening. Our results showed that a total of 32 proteins were differentially expressed, with 16 of them showing increased expression and the other 16 showing decreased expression in the SiHa cells in the presence of AS-IV. It is suggested that AS-IV is different from smaller macromolecular compounds and has no multiple “target holes” in the body. The target of AS-IV may be among these 32 differentially expressed proteins. Subsequently, we assessed the 32 differentially expressed proteins with respect to their BPs, CCs, and MFs. The results showed that the proteins are regulatory proteins controlling cell proliferation and development. While the CC of these proteins are actin–myosin complexes, they perform MFs such as steroid binding and altering the composition of the cell cytoskeleton. The role of PRM is to validate the proteomic data of iTRAQ, just as using q-PCR to validate transcriptome data. Therefore we selected several key differentially expressed proteins from iTRAQ proteomic data, such as protein DCP1A, TMSB4X, MGST3, AKR1C2, ERLIN1, and then obtained more accurate quantitative values of these proteins by PRM. We found that five of these differentially expressed proteins showed the same expression tendency as that inferred through iTRAQ analysis (Fig. 5a). Our results showed that the protein expression of DCP1A and TMSB4X increased in cervical cancer cells in the presence of AS-IV. Our results also showed that the protein expression of MGST3, AKR1C2, and ERLIN1 decreased in cervical cancer cells in the presence of AS-IV. We also found that AS-IV could target the proteins DCP1A and TMSB4X and induce autophagy in suppressing cervical cancer invasion. The function of the five proteins is related to the progress of tumor cell biology. Out of these five proteins, DCP1A regulates Atg12 and is linked to autophagy and proteasome degradation pathways [40], TMSB4X regulates TGFβ1 and participates in the progression of cancer [41], MGST3 increases in the LT97 adenoma cells and participates in cancer development [42], AKR1C2 is a key enzyme in the process of progesterone metabolism and is closely related to gynecological tumor development [43], and ERLIN1 is influences the survival time of pancreatic cancer patients [44].

In summary, we performed iTRAQ combination by PRM to identify the potential target of AS-IV in the process of suppressing cervical cancer cell invasion. AS-IV could inhibit cervical cancer invasion by inducing autophagy. While DCP1A and TMSB4X may be regarded as targets of AS-IV in the process of autophagy, they also may be regarded as targets in the screening of anticancer compounds used in the treatment of cervical cancer. Thus, iTRAQ combination by PRM can be used in the identification of macromolecular target compounds and can also be considered as a novel technique in the screening of anticancer compounds used in the treatment of cervical cancer.

## Supplementary information

**Additional file 1.**

## Data Availability

Not applicable.

## Abbreviations

AS-IV: Astragaloside IV
iTRAQ: Isobaric tags for relative and absolute quantitation
PRM: Parallel reaction monitoring
DCP1A: mRNA-decapping enzyme 1A
TMSB4X: Thymosin beta-4
MGST3: Microsomal glutathione S-transferase 3
AKR1C2: Aldo-keto reductase family 1 member C2
