# Supplementary material for: Quantitative proteomics analysis of differentially expressed proteins induced by astragaloside IV in cervical cancer cell invasion
Source: Cell Mol Biol Lett. 2020 Mar 31;25:25. doi: 10.1186/s11658-020-00218-9 (PMC7110762; doi:10.1186/s11658-020-00218-9)

## Slide 1
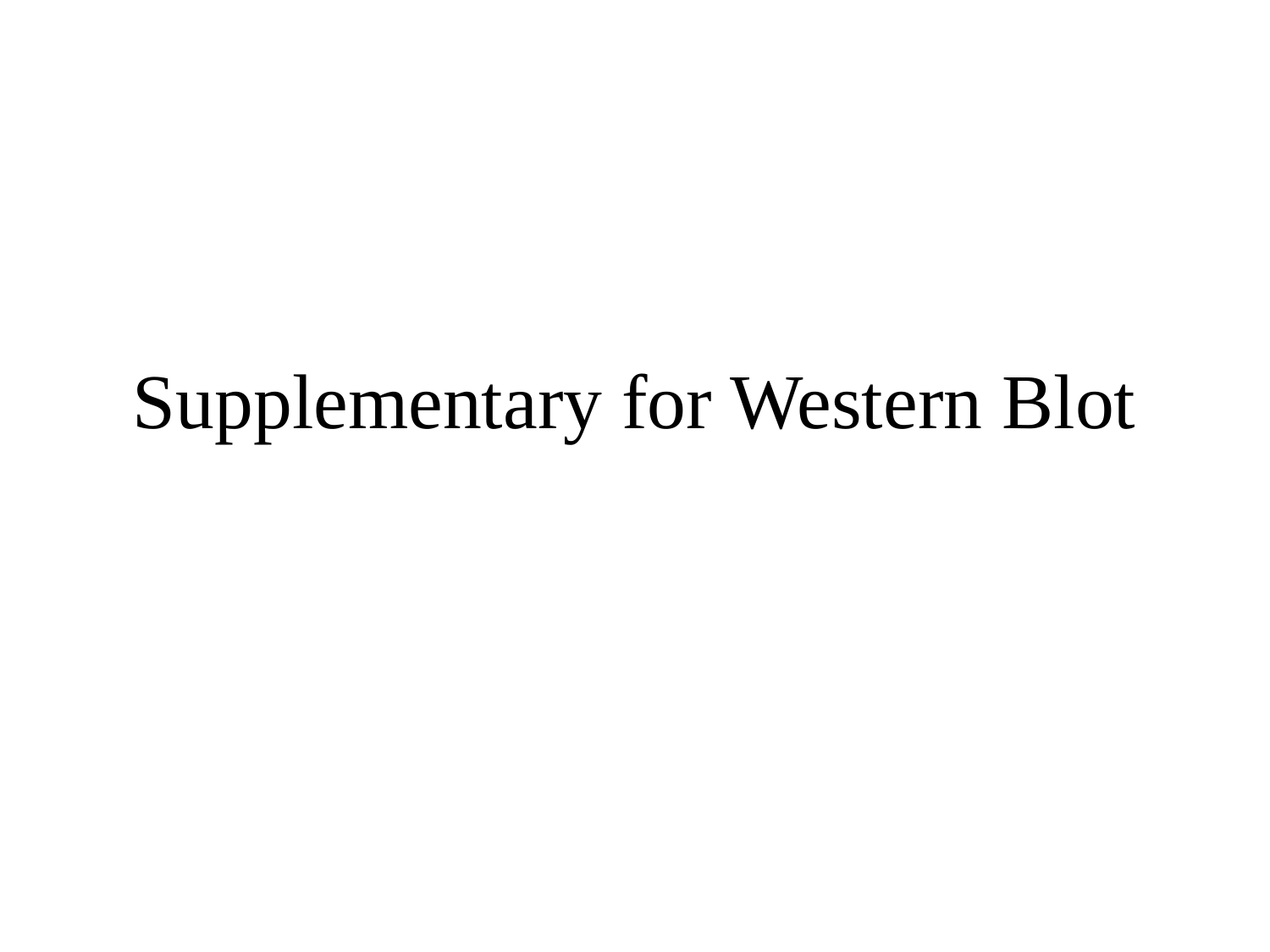

# Supplementary for Western Blot

## Slide 2
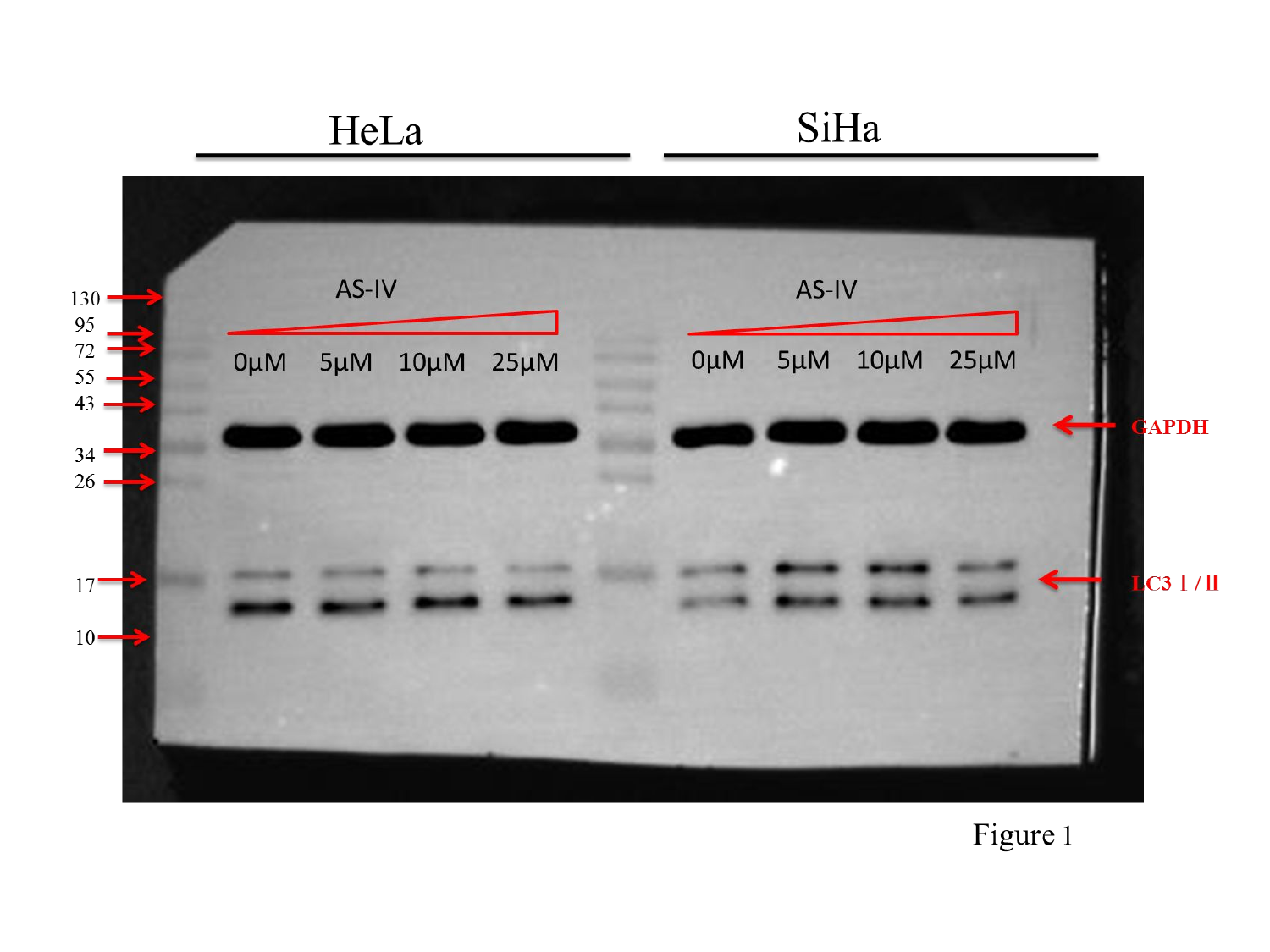

## Slide 3
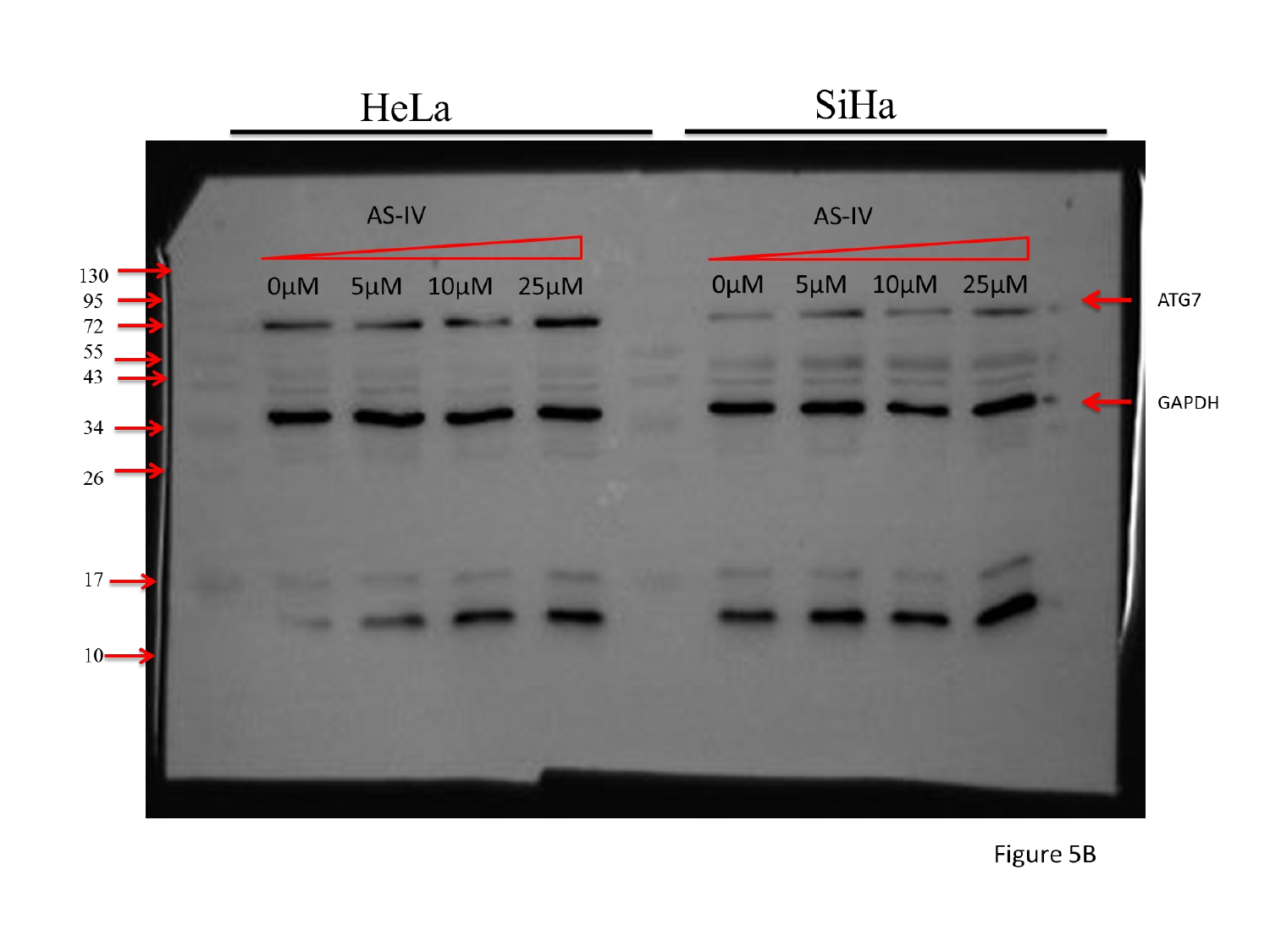

## Slide 4
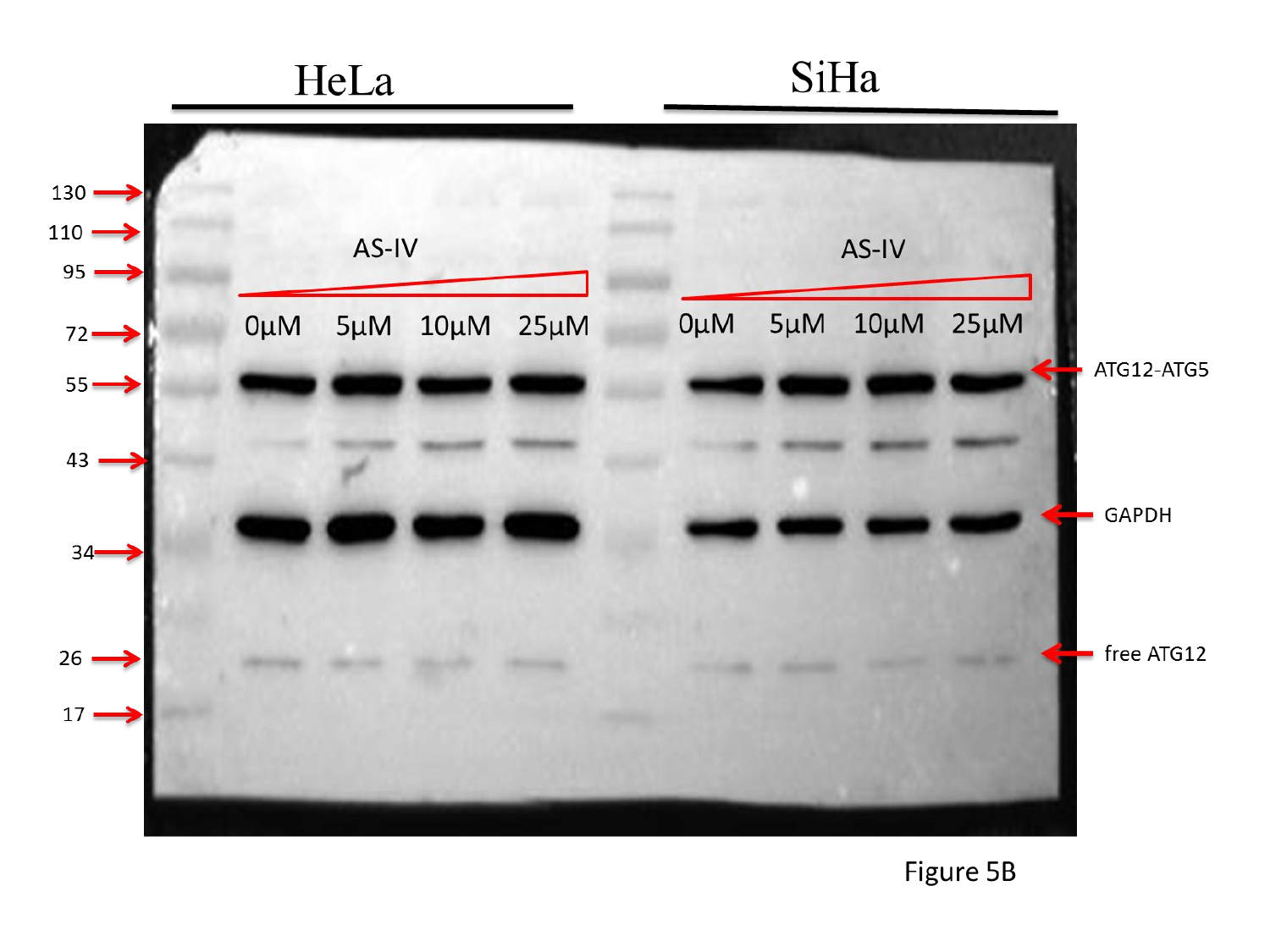

## Slide 5
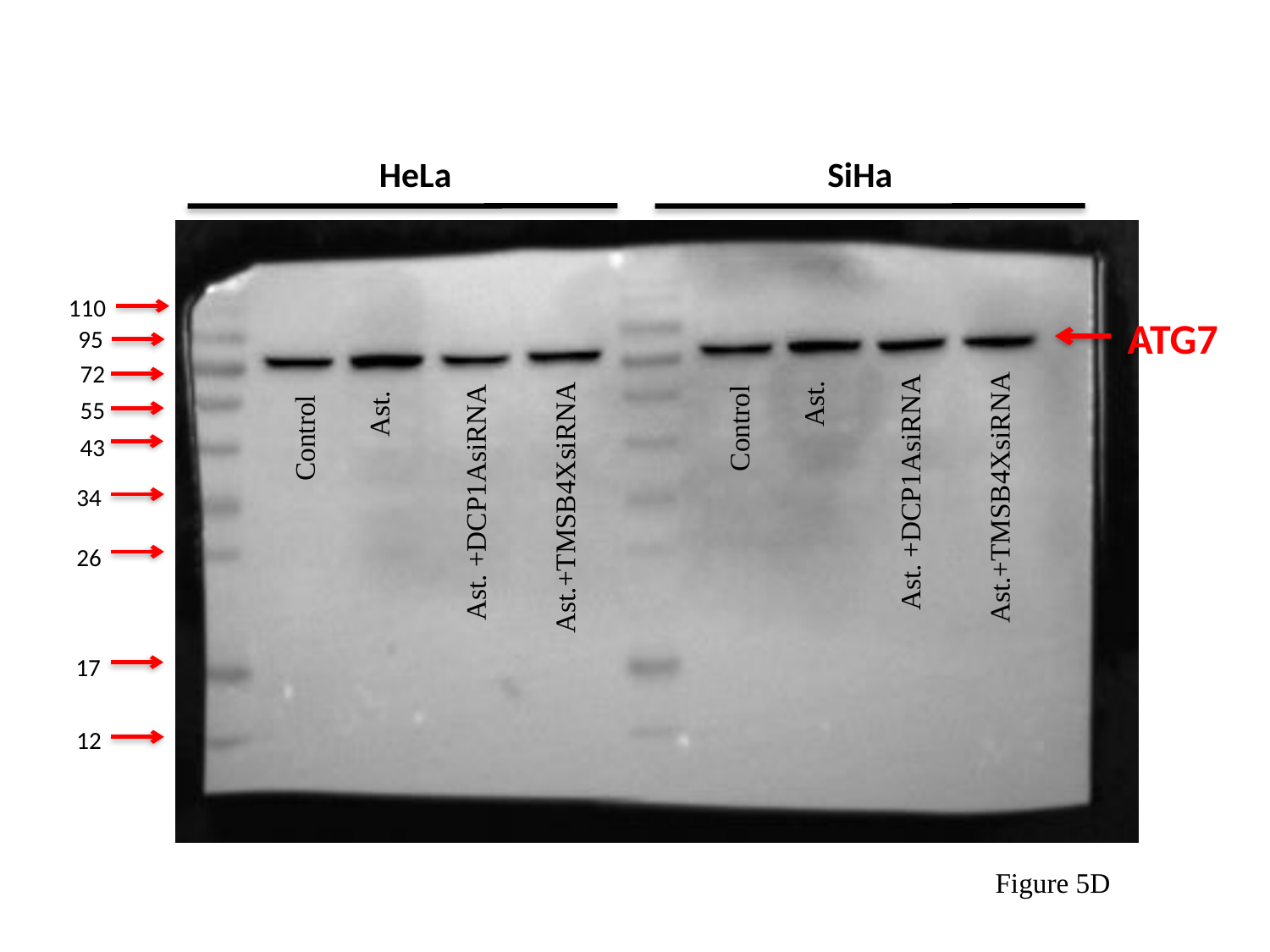

HeLa
SiHa
110
95
72
55
43
34
26
17
12
ATG7
Ast.
Ast.
Control
Control
Ast. +DCP1AsiRNA
Ast.+TMSB4XsiRNA
Ast. +DCP1AsiRNA
Ast.+TMSB4XsiRNA
Figure 5D

## Slide 6
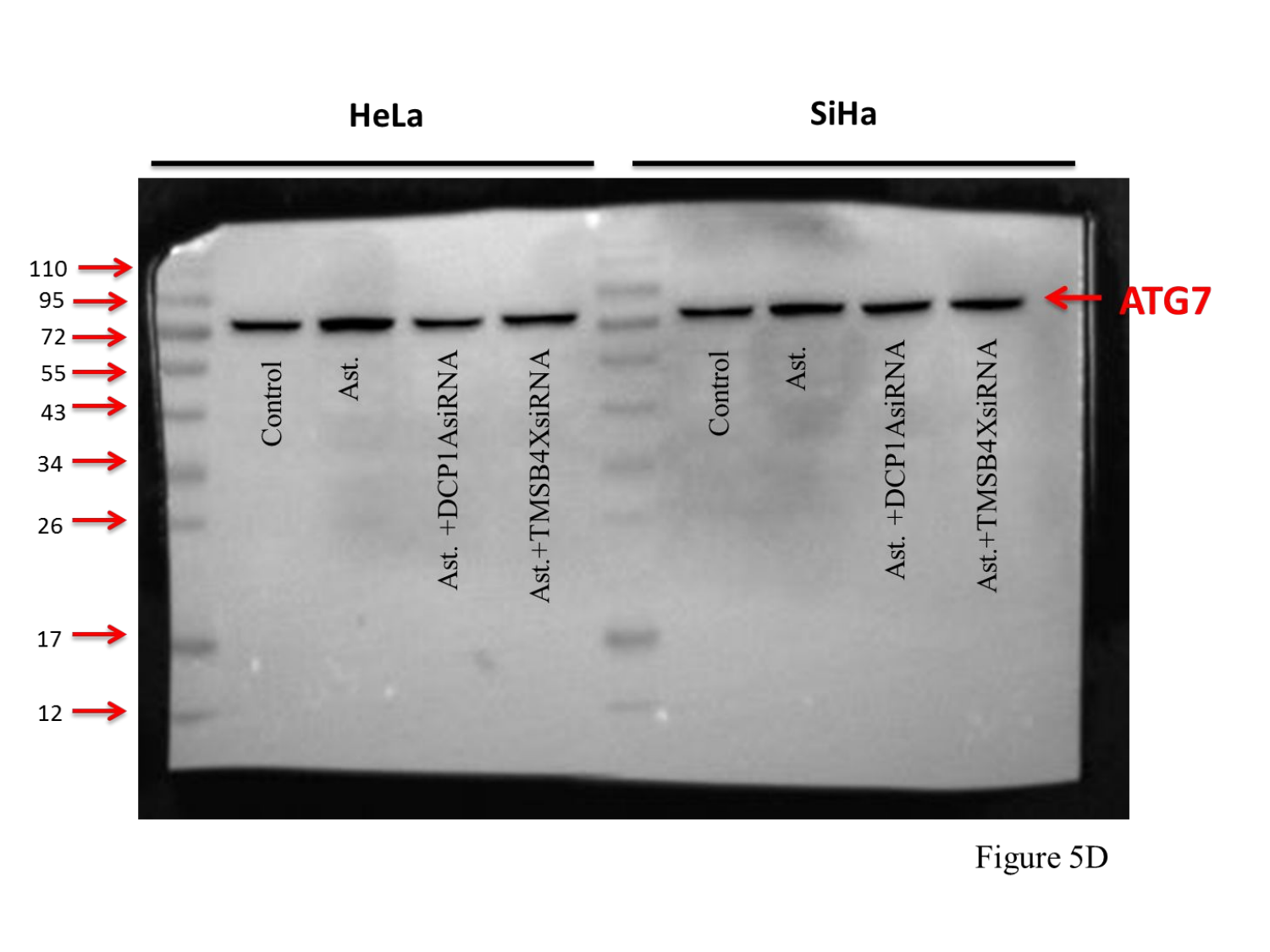

## Slide 7
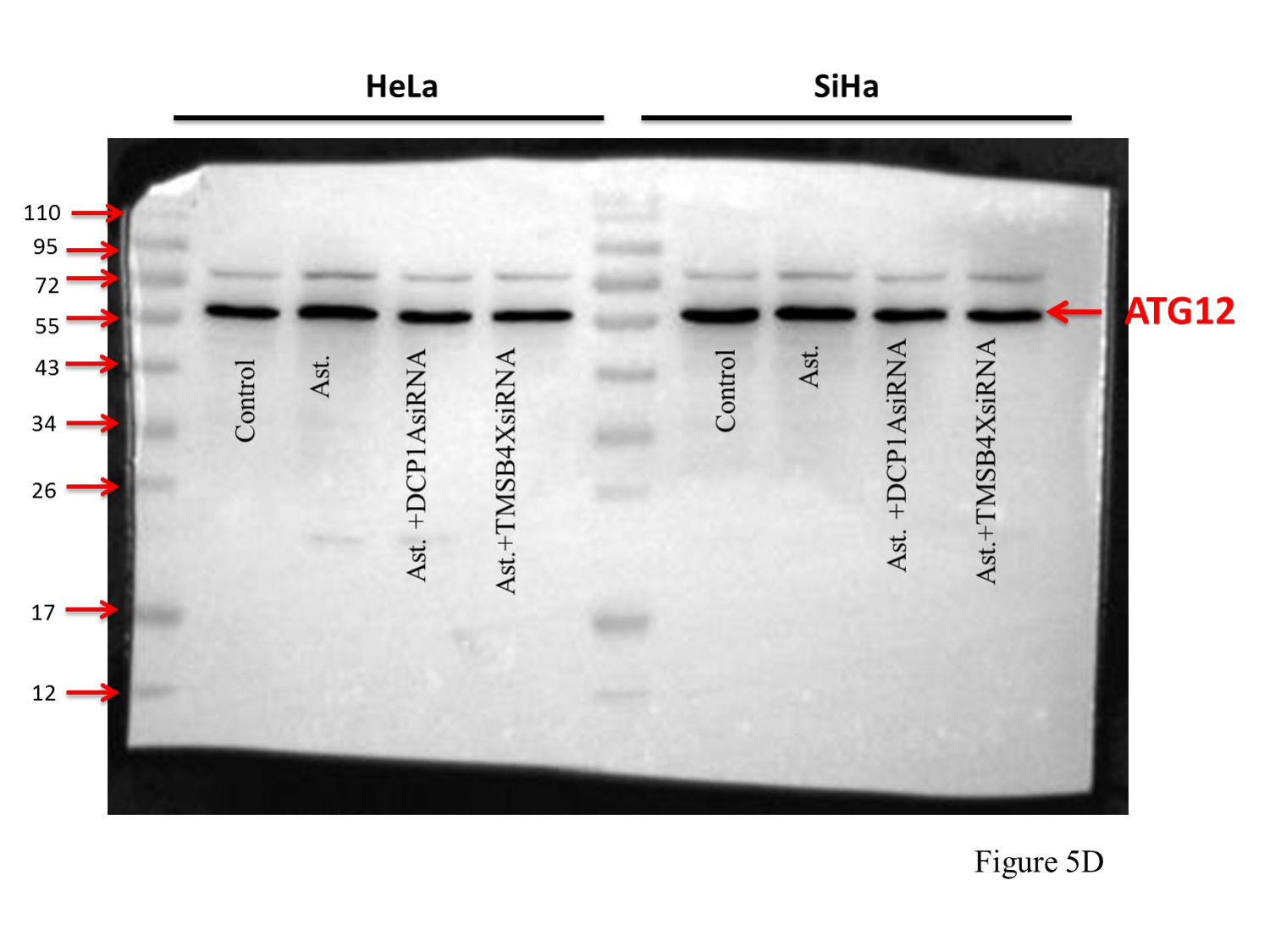

## Slide 8
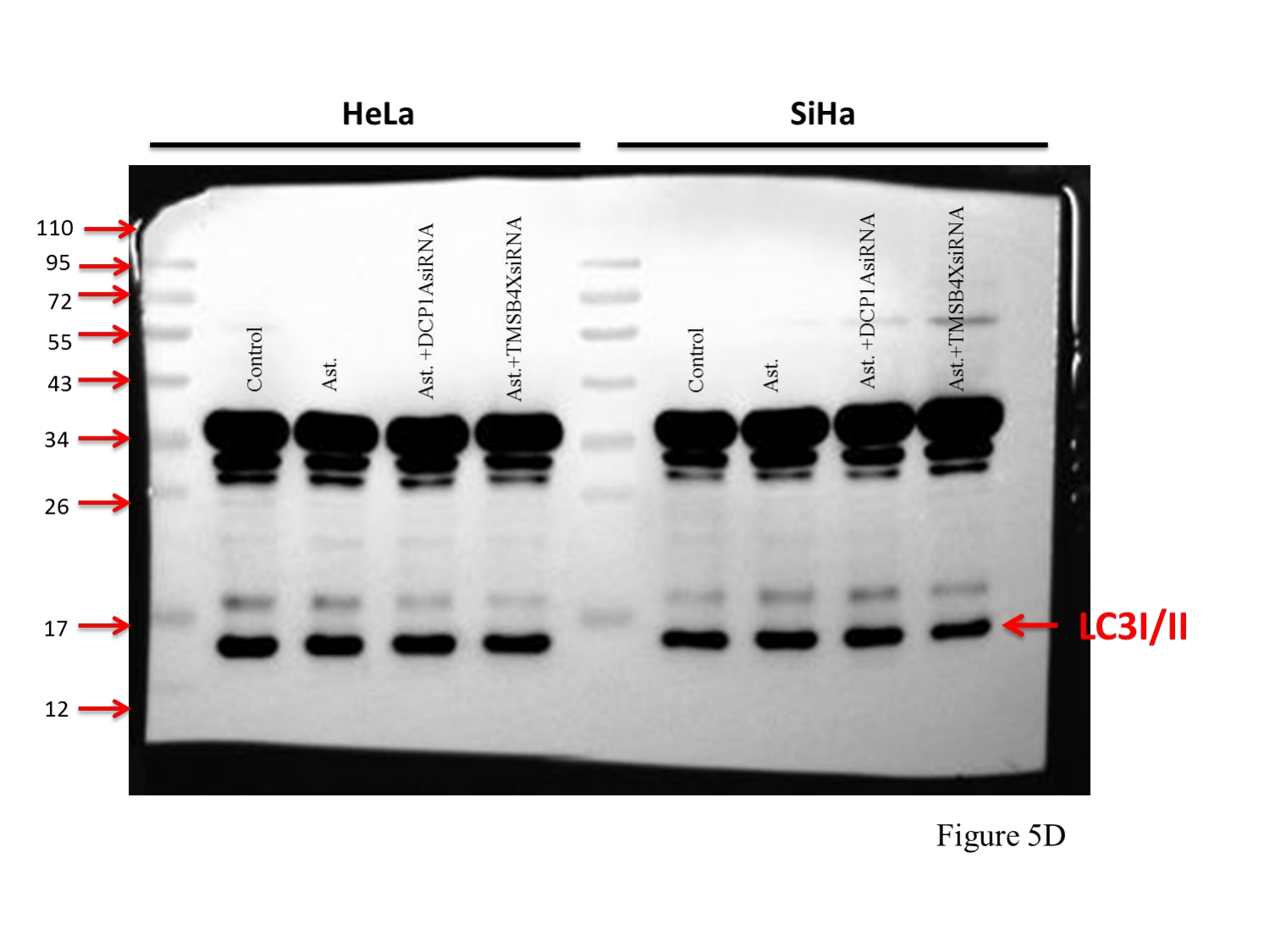

## Slide 9
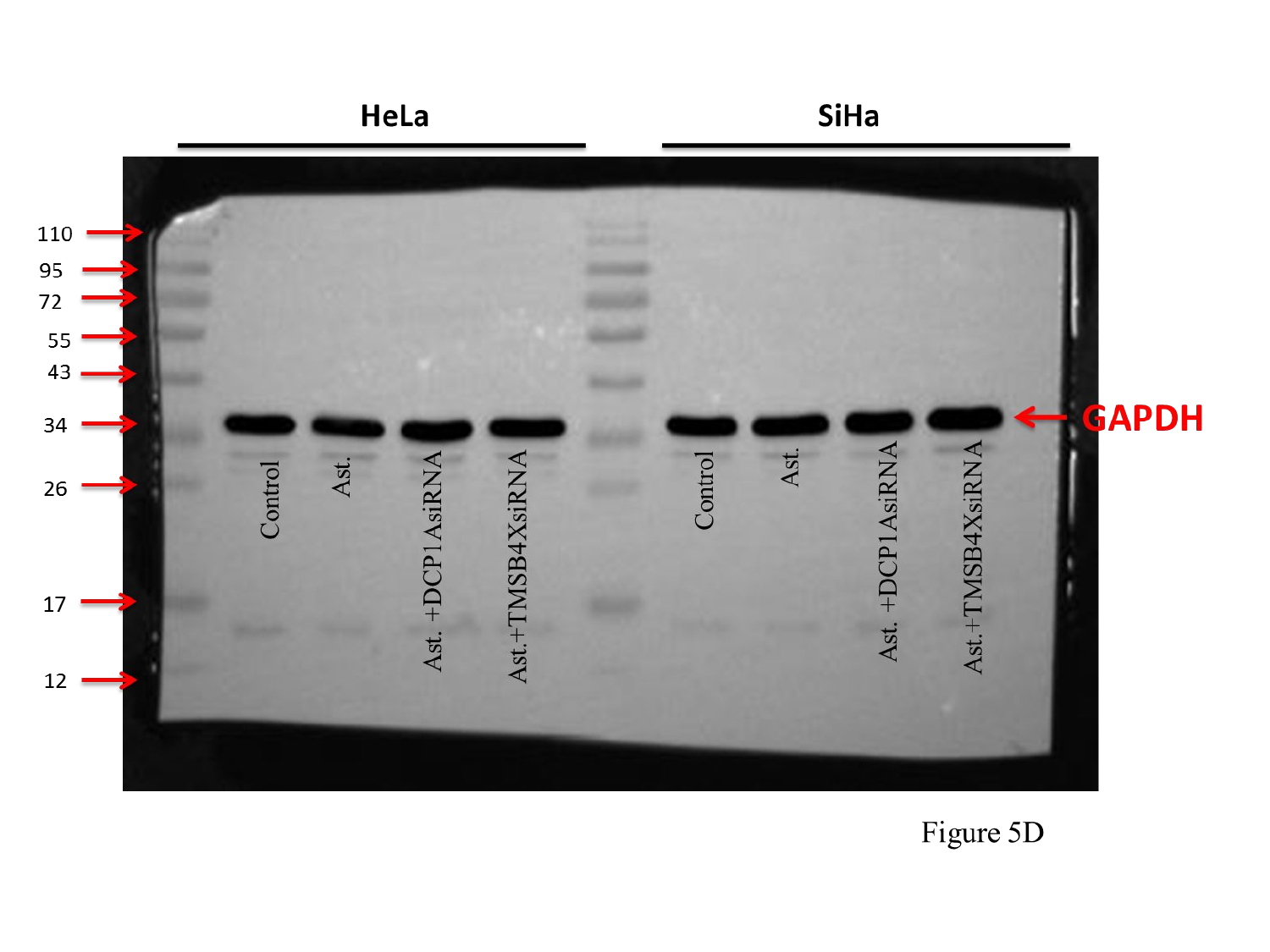

## Slide 10
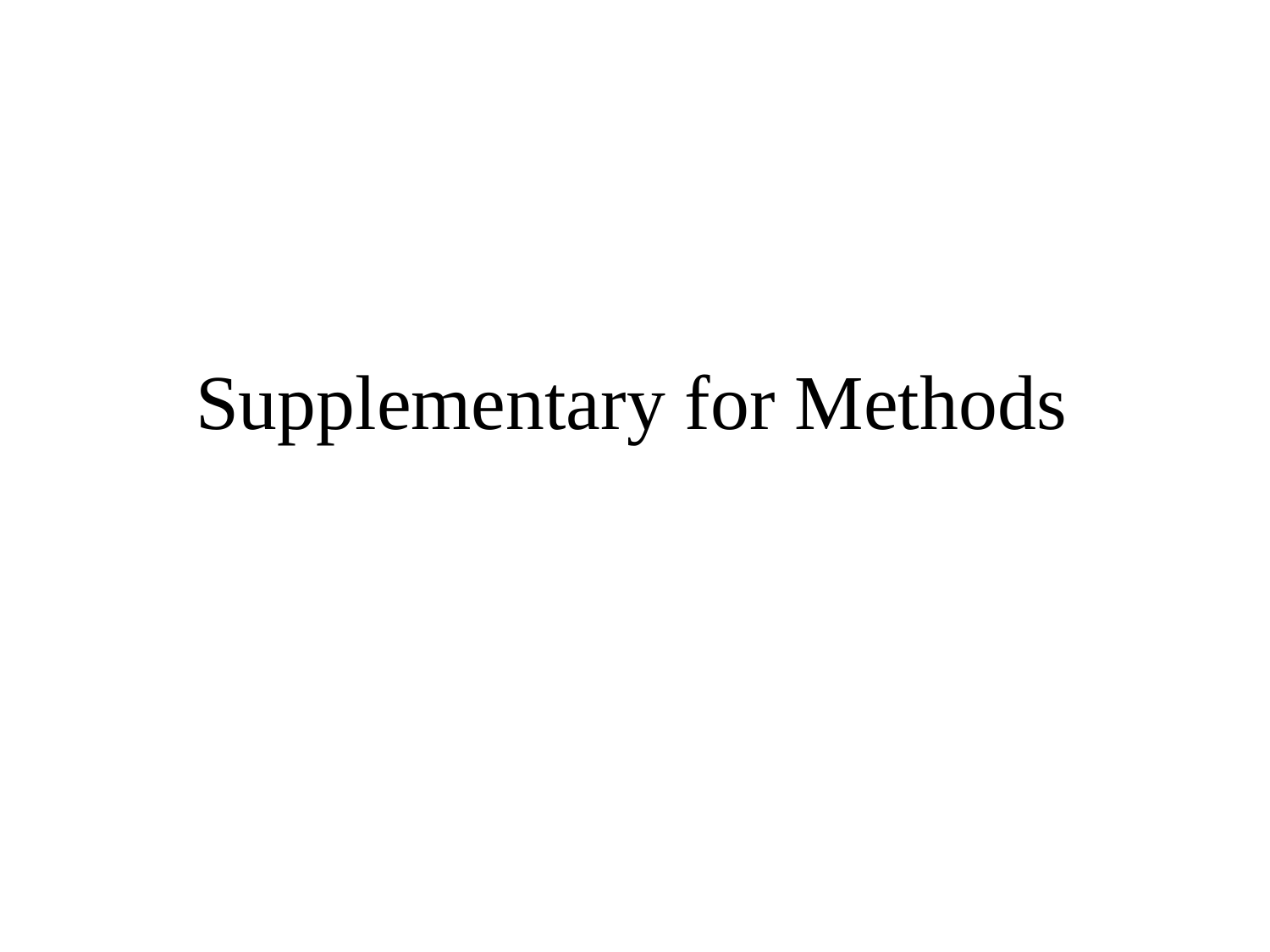

# Supplementary for Methods

## Slide 11
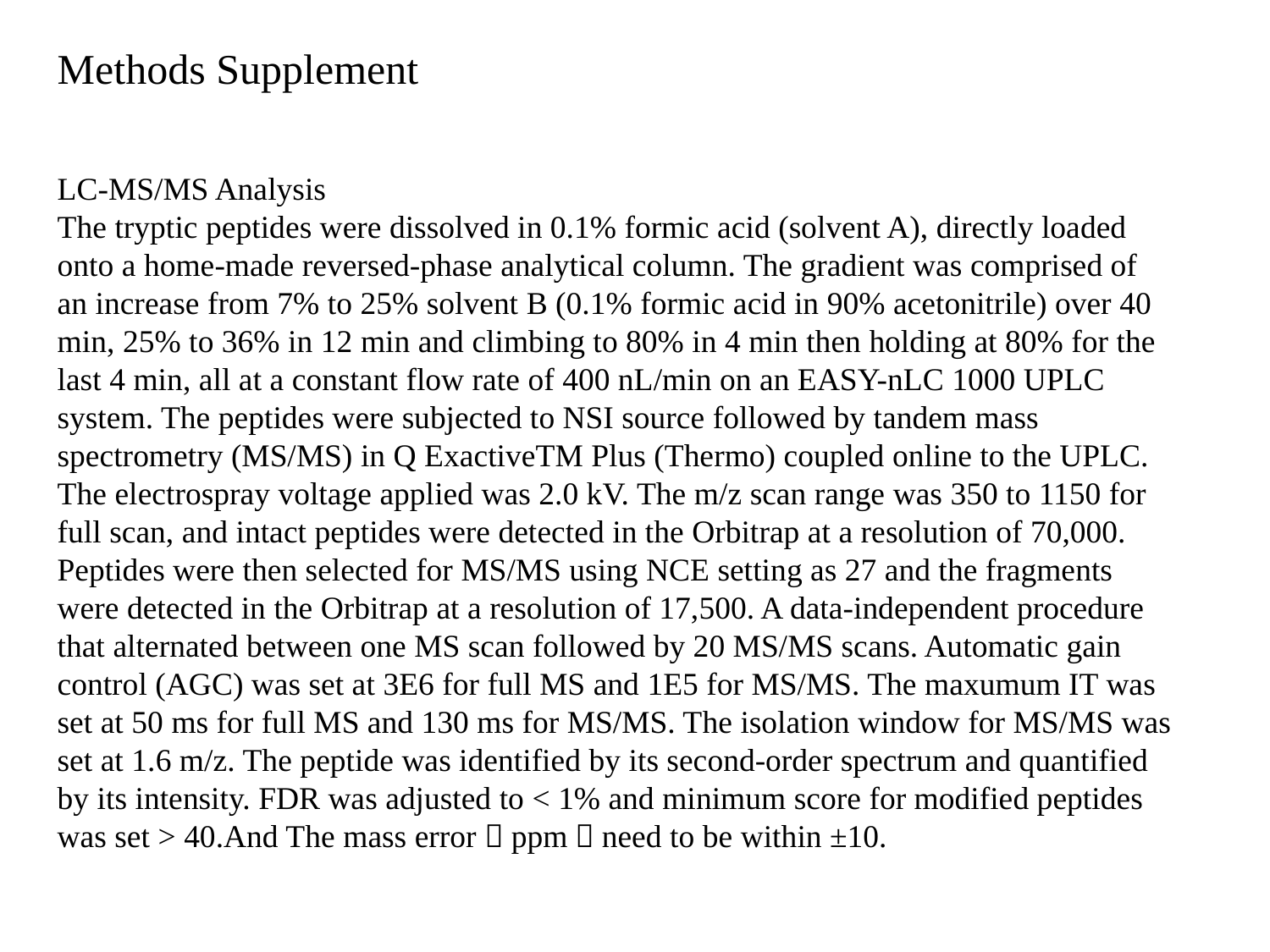

Methods Supplement
LC-MS/MS Analysis
The tryptic peptides were dissolved in 0.1% formic acid (solvent A), directly loaded onto a home-made reversed-phase analytical column. The gradient was comprised of an increase from 7% to 25% solvent B (0.1% formic acid in 90% acetonitrile) over 40 min, 25% to 36% in 12 min and climbing to 80% in 4 min then holding at 80% for the last 4 min, all at a constant flow rate of 400 nL/min on an EASY-nLC 1000 UPLC system. The peptides were subjected to NSI source followed by tandem mass spectrometry (MS/MS) in Q ExactiveTM Plus (Thermo) coupled online to the UPLC. The electrospray voltage applied was 2.0 kV. The m/z scan range was 350 to 1150 for full scan, and intact peptides were detected in the Orbitrap at a resolution of 70,000. Peptides were then selected for MS/MS using NCE setting as 27 and the fragments were detected in the Orbitrap at a resolution of 17,500. A data-independent procedure that alternated between one MS scan followed by 20 MS/MS scans. Automatic gain control (AGC) was set at 3E6 for full MS and 1E5 for MS/MS. The maxumum IT was set at 50 ms for full MS and 130 ms for MS/MS. The isolation window for MS/MS was set at 1.6 m/z. The peptide was identified by its second-order spectrum and quantified by its intensity. FDR was adjusted to < 1% and minimum score for modified peptides was set > 40.And The mass error（ppm）need to be within ±10.

## Slide 12
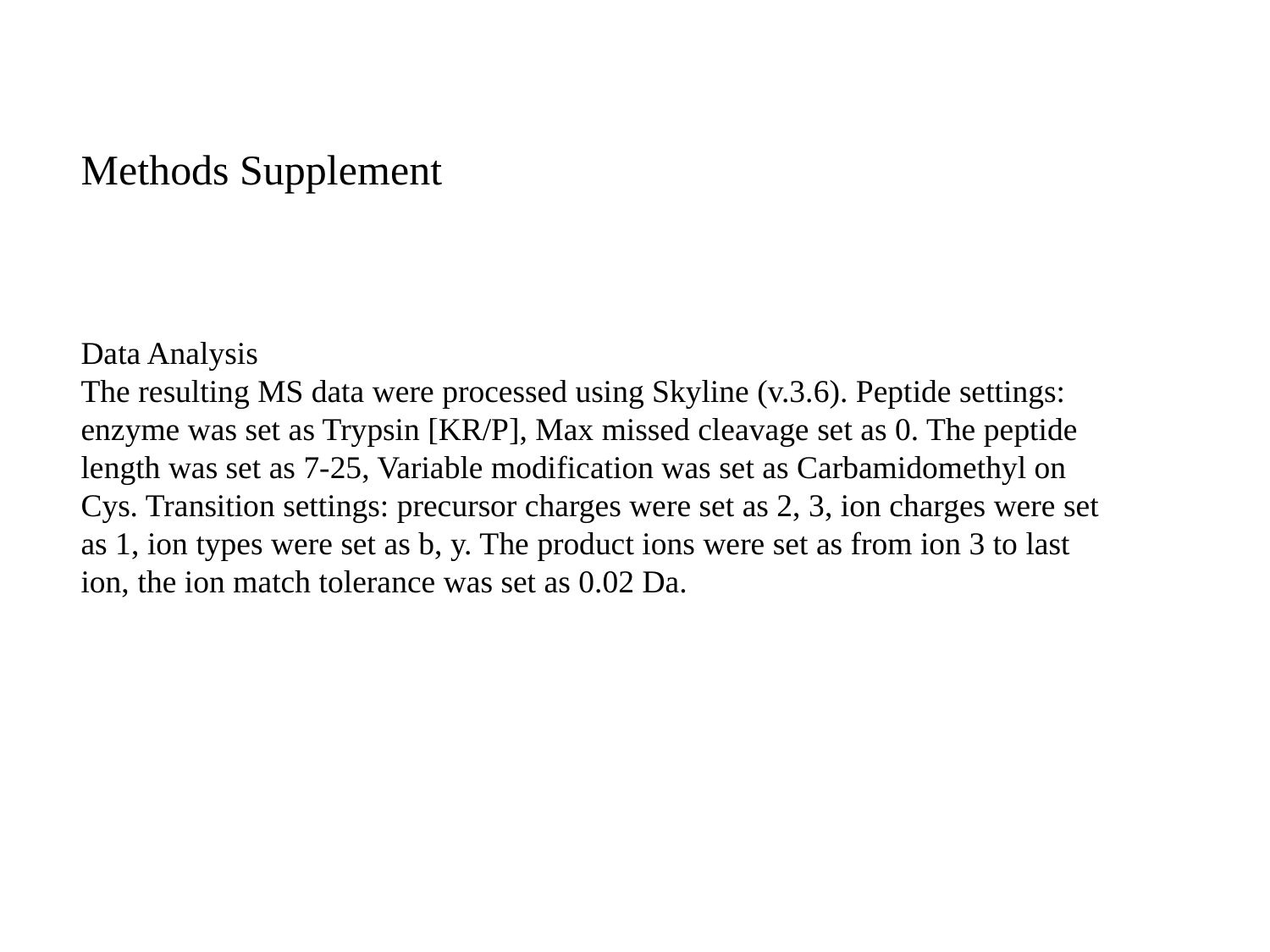

Methods Supplement
Data Analysis
The resulting MS data were processed using Skyline (v.3.6). Peptide settings: enzyme was set as Trypsin [KR/P], Max missed cleavage set as 0. The peptide length was set as 7-25, Variable modification was set as Carbamidomethyl on Cys. Transition settings: precursor charges were set as 2, 3, ion charges were set as 1, ion types were set as b, y. The product ions were set as from ion 3 to last ion, the ion match tolerance was set as 0.02 Da.

## Slide 13
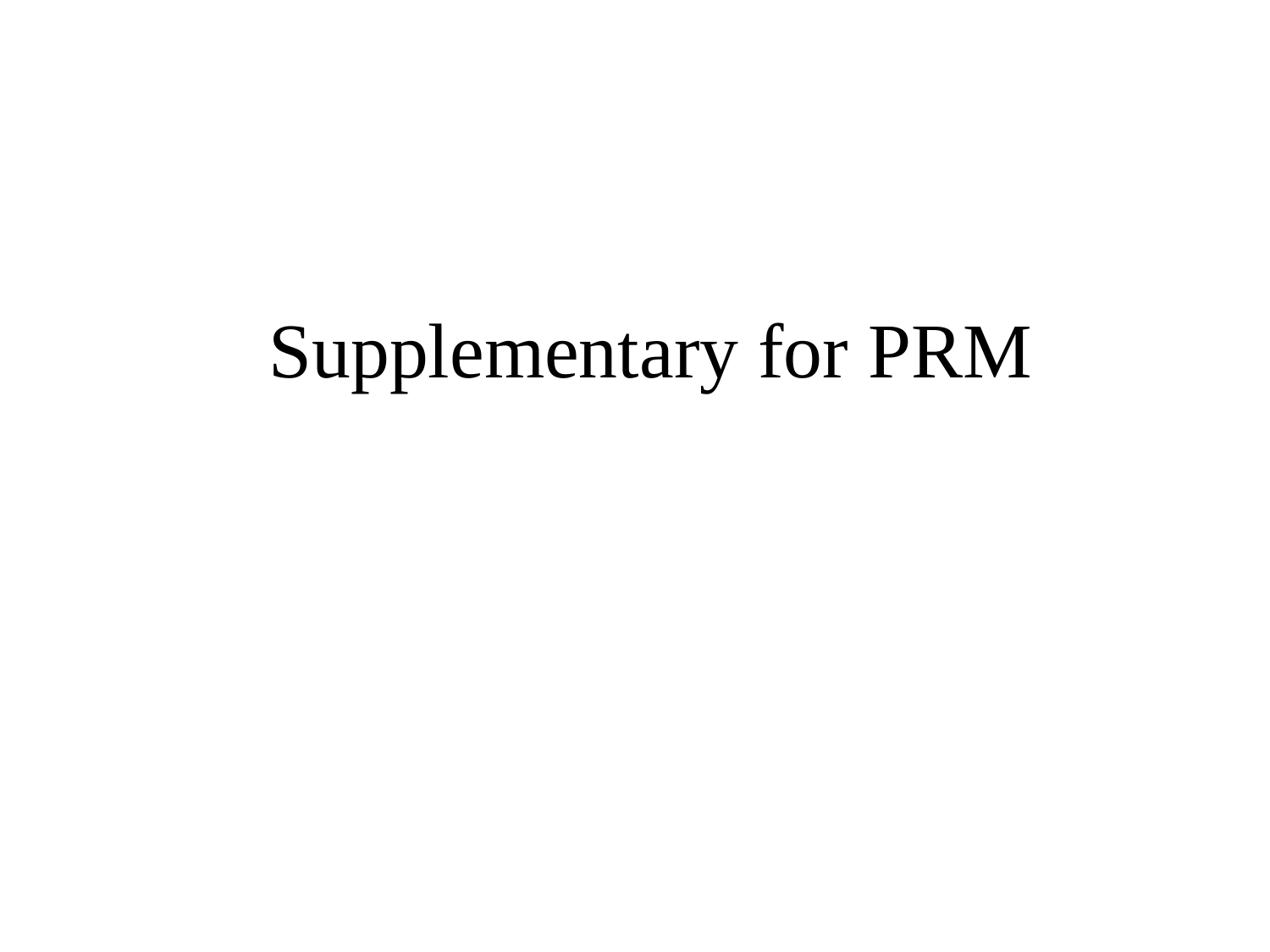

# Supplementary for PRM

## Slide 14
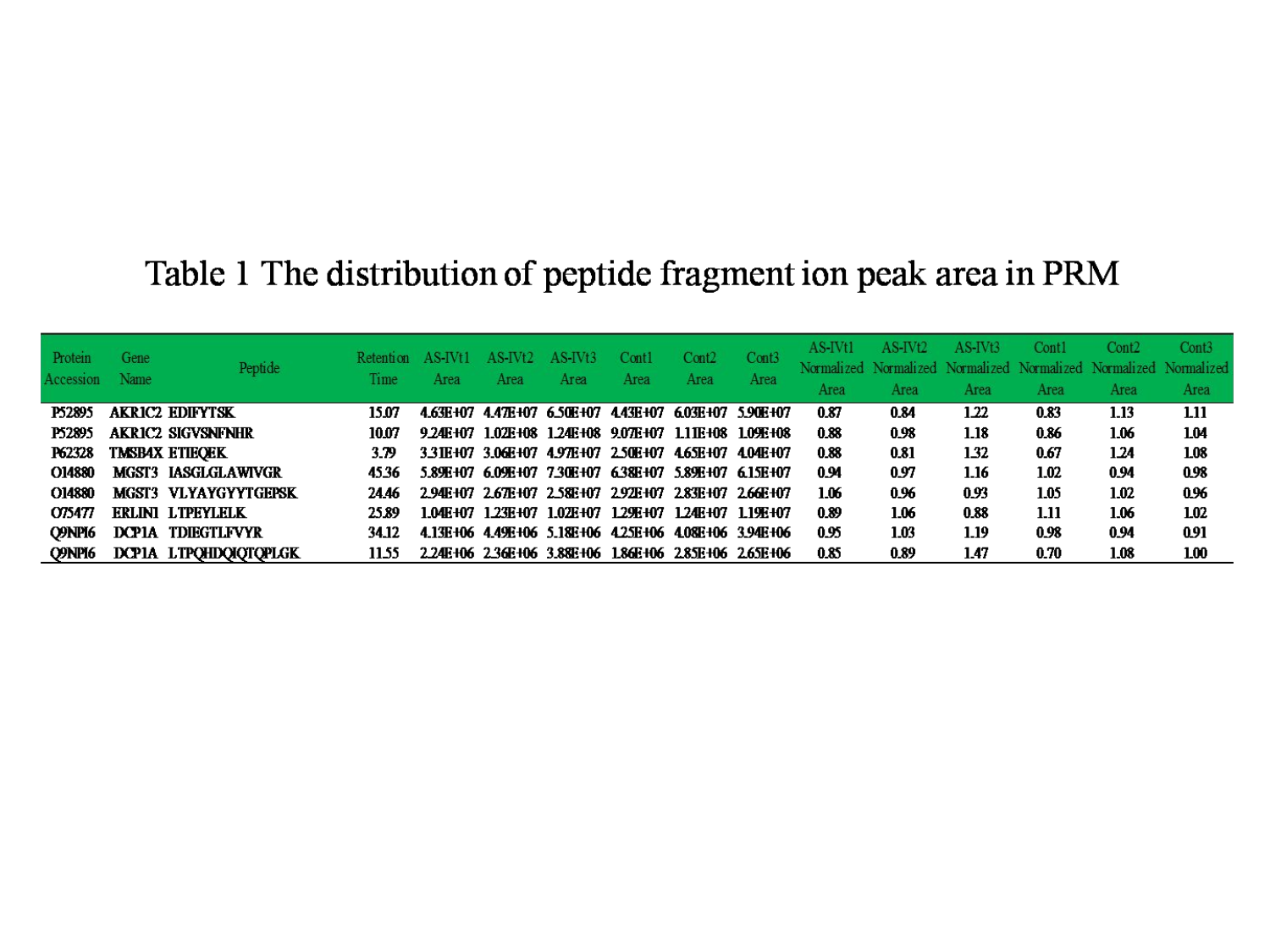

Supplement: Supplementary file 1 — Additional file 1. [file 11658_2020_218_MOESM1_ESM.pptx]
